# Integrated Behavior Therapy for Exclusively Anxious Selective Mutism: A Nonconcurrent Multiple-Baseline Design across Five Participants

**DOI:** 10.3390/pediatric15040057

**Published:** 2023-10-16

**Authors:** Allison K. Siroky, John S. Carlson, Aimee Kotrba

**Affiliations:** 1 Nationwide Children’s Hospital Big Lots Behavioral Health Services, Columbus, OH 43215, USA; allison.siroky@nationwidechildrens.org; 2 Department of Counseling, Educational Psychology & Special Education, College of Education, Michigan State University, East Lansing, MI 48224, USA; 3 Thriving Minds Behavioral Health Center, Brighton, MI 48116, USA; akotrba@gmail.com

**Keywords:** selective mutism, behavioral therapy, manualized treatment, social anxiety, children, social phobia, adherence, effectiveness, acceptability

## Abstract

Selective mutism (SM) is a rare childhood anxiety disorder which may be markedly detrimental to a child’s academic and social functioning if left untreated. Cognitive–behavioral treatments for social anxiety disorders have been found to be effective for SM, yet a paucity of published studies have explored manualized treatment approaches carried out by novice clinicians. The purpose of the present study was to examine the adherence, effectiveness, and acceptability of a condensed, 16-session version of Integrated Behavior Therapy for Selective Mutism (IBTSM; Bergman, 2013), the first manualized treatment for SM. A nonconcurrent multiple-baseline single-case design was used across five children diagnosed with SM, exclusively anxious subtype. IBTSM was implemented with excellent adherence (M = 98%) over an average of 19 weeks (range = 16–22 weeks). Visual analyses of weekly caregiver ratings of social anxiety and speaking behaviors did not demonstrate a replicated intervention effect; however, Tau-U effect sizes and Reliable Change Index (RCI) calculations demonstrated significant individual improvements in social anxiety and speaking behaviors over time on several measures. Three children (60%) no longer met diagnostic criteria for SM following treatment. All caregivers rated IBTSM as acceptable, with specific endorsements of acceptability in the areas of time required and treatment quality.

## 1. Introduction

Selective mutism (SM) is a rare anxiety disorder in which children persistently fail to speak in certain social settings (e.g., at school, in public), though they exhibit comfortable and consistent speech in others (e.g., at home, with caregivers, close friends). Despite its low prevalence rate, with less than two percent of children receiving a diagnosis of SM [1], the potentially debilitating effects of SM on later development [2,3] create a need for increased awareness of SM, including efforts to promote earlier diagnostic identification and wider dissemination of potential evidence-based treatment (EBT) options for children with this disorder. Research on the epidemiology of SM strongly suggests that SM symptoms are likely a result of excessive anxiety when faced with an expectation to speak in novel situations [4,5]. The reclassification of SM as an anxiety disorder in the fifth edition of the Diagnostic and Statistical Manual for Mental Disorders (DSM-5) [6] further highlights the association between SM and social anxiety.

Psychosocial treatment approaches (e.g., cognitive–behavioral therapy, behavioral therapy) are currently supported as the most effective option for both social anxiety and SM [1,7,8,9]. Given the young age of onset (i.e., typically before the age of five years), behavioral therapy may be most appropriate for this young population [10]. Behavioral therapy for SM typically involves similar behavioral techniques to the treatment of social phobia such as psychoeducation about anxiety, graduated exposures, and a reward plan to reinforce the successful completion of exposure tasks [11,12]. Unfortunately, the rarity of SM makes it likely that clinicians have limited experience in assessing or treating children with SM, highlighting the need for manualized EBTs especially in areas with limited access to trained clinicians. Further complicating the treatment of SM is the need to consider the impact of SM subtypes including those identified as multifaceted anxiety, oppositional, and/or communication-related [13].

Bergman [10] sought to address this noticeable gap in the current state of evidence-based practice for SM by developing Integrated Behavior Therapy for Selective Mutism (IBTSM), a 24-week manualized treatment program for children ages four to eight years diagnosed with SM. Through a randomized controlled trial (RCT), 25% of children who received IBTSM no longer met criteria for SM at the midpoint assessment and two-thirds (67%) saw a removal of diagnosis by the end of treatment [14]. Study findings supported further examination of IBTSM within community-based settings with consideration to whether a modified treatment length could produce comparable gains.

Two additional studies have explored the effectiveness of IBTSM with modifications to treatment length and/or the setting implemented. The first study [15] condensed IBTSM to 12 sessions over 18 weeks within a replicated case study design. Notably, the two participants presented with different characteristics and associated symptoms (i.e., one with comorbid social anxiety disorder and the other with elevated oppositional behaviors). Although both participants showed improved speaking behaviors and reductions in caregiver-reported social anxiety levels by the end of treatment, only the child with primarily anxious presentation at baseline no longer met criteria for SM by the three-month follow-up. A second study [16] used a 35-session version of IBTSM to treat a five-year-old boy with SM, separation anxiety, and comorbid speech/language deficits. The length of IBTSM was extended to allow for continued services while the child underwent a speech/language evaluation. Parent ratings indicated that this participant’s symptoms of symptoms of anxiety, depression, and withdrawal all fell within normal limits, as compared to same-age peers, by the end of IBTSM.

These early investigations provide some indication of the potential transportability and effectiveness of the manualized IBTSM for treating children with SM in applied settings. Further study is needed, however, to better understand for which subtypes of SM, and under which conditions, IBTSM may be consistently effective. Reviews of the SM treatment literature agree that behavioral therapy is generally effective for samples of children with SM [1,12], but Cohan and colleagues [17] argue that differing presentations of SM may benefit from more targeted treatment approaches. Scholars of SM are continuing to explore potential subtyping classification systems for SM, given that some children with SM also present with autism spectrum problems, oppositionality, or have a comorbid speech/language disorder [1,6].

Cohan and colleagues [17] identified three subtypes of SM, distinguishing between those who are exclusively anxious, those with mild oppositional behaviors and anxiety, and those with a speech/language disorder or communication delay and anxiety as well. They argued these diverse clinical profiles required distinct treatment approaches, suggesting that behavioral therapy alone may be ideal for children who present with the proposed subtype of exclusively anxious SM, while children with mild-oppositional/anxious tendencies might benefit from an approach that includes a more structured contingency management plan to reinforce compliance. Alternatively, children with a comorbid communication-delay would likely need speech therapy, along with behavioral therapy.

This call for subtype-informed treatment aligns with recent trends in clinical practice that seek to distill specific components of EBTs and analyze their effectiveness to match client characteristics [18]. However, the absence of any EBT designed specifically for children with SM [19] makes it difficult for clinicians who are less familiar with this disorder to use such a distillation and matching process to address the individual needs of the children and families they serve. A manualized treatment approach such as IBTSM may be ideal for this purpose to ensure replicability when implemented in new contexts among a range of clinicians and with clearly specified populations (i.e., subtypes) of youth with SM.

The purpose of the current study was to examine the adherence, effectiveness, and acceptability of condensed version of IBTSM (i.e., 16 sessions) administered in a community-based clinical setting by novice clinicians with five children identified as presenting with exclusively anxious SM. Four primary research questions guided this study: (1) Can IBTSM be carried out as intended by novice clinicians in a community-based clinical setting across all five cases? (2) Would IBTSM lead to a meaningful decrease in caregiver-rated social anxiety levels across the baseline phases and treatment phases? (3) Would IBTSM lead to meaningful improvements in observed speaking behaviors across settings? (4) Would IBTSM be rated by caregivers as an acceptable treatment approach?

## 2. Materials and Methods

### 2.1. Participants

Five children (ages 4–8 years; M = 6.2 years) were enrolled as participants in the present study (see Table 1 for additional demographics). In order to be eligible to participate, children had to have been between the ages of four and eight years at the time of recruitment, per suggestions from the manual’s developer [10]. All five participants presented with symptoms consistent with a diagnosis of SM, as confirmed by the baseline clinical interview. Due to early research suggesting that behavioral therapy may be most appropriate for children with the proposed subtype of exclusively anxious SM, children eligible for this study also needed to demonstrate symptoms consistent with an exclusively anxious presentation of SM [17]. Baseline data indicated that all five participants demonstrated stable symptoms (e.g., persistently low frequency of speaking behaviors across settings) during the baseline phase. Finally, because IBTSM does not incorporate strategies for eliciting speech in very severe SM cases, participants were screened for moderate SM severity, as indicated by a total score on the Selective Mutism Questionnaire (SMQ) [20] between 13 and 27 (i.e., mean for children with SM plus two standard deviations above the mean). All children presented with elevated symptoms of social anxiety at baseline, as rated by caregivers using the (Social Anxiety Scale for Children—Revised; SASC-R, Parent Version) [21].

To avoid potential conflicting or invalid results for this treatment effectiveness study, participants must not have been receiving any other form of treatment for SM at the time of enrollment, including other psychosocial treatment approaches or psychopharmacological treatment. Although not a formal inclusion criterion, it was recommended that participants be enrolled in a full-time school program (e.g., preschool, early childhood center, elementary school) for the entirety of the study [10]. Four of five participants were enrolled in full-time school for a majority of the time they were in treatment, and the fifth participant (Child 5) was enrolled in part-time preschool for three afternoons per week.

### 2.2. Study Design

This study used a nonconcurrent multiple-baseline single-case design, following an AB series. with data collection occurring at different times for all five families. Baseline lengths ranged from five days to nine days. Randomization (i.e., randomly assigning baseline lengths to each participant) was not utilized due to limitations in parent and clinician scheduling; however, baseline lengths were controlled so that no two families could have the same treatment length.

#### 2.2.1. Recruitment and Consent/Assent Procedures

Participants for the present study were recruited primarily through the participating community-based psychology clinic in mid-Michigan. Families who expressed interest in the present study received information about the expectations for participation, including where weekly sessions would occur. Those who continued to be interested received an initial screening packet. A total of 13 families contacted the study coordinator to express interest. Of these, seven families (53.8%) moved on to the screening process to determine eligibility, while the others (n = 6; 46.2%) were not eligible due to distance (i.e., lived out of state, lack of availability in schedule to travel to clinic) or initial exclusion criteria (e.g., recently enrolled in intensive-dose behavioral therapy for SM). All recruitment and active treatment activities took place prior to the COVID-19 pandemic, so in-person sessions were required. Following screening with the remaining seven families, the first five children who met all eligibility criteria were enrolled.

Parental consent was obtained at the parent-only pre-treatment intake session. An additional child assent form was administered to the child at the beginning of the first therapy session. Clinicians were encouraged to obtain verbal assent from the child but, given expected difficulties with children speaking at baseline, options for nonverbal assent (e.g., pointing, nodding) were provided as well. All families received one pre-treatment intake session and 16 active treatment sessions at no cost, with an additional USD 200 in compensation for their active participation and to reduce barriers to treatment attendance (e.g., cost travel to/from the clinic, lack of toys/rewards for contingency management).

#### 2.2.2. Baseline Phase

After the initial screening procedures and the parent-only pre- treatment session, families who were invited to participate and who provided their consent transitioned to the baseline phase. The minimum baseline length was five days and the maximum length was nine days so as to avoid excessive or unnecessary delays in providing treatment to participants. Baseline assignments were determined primarily by the family and the clinician’s availability. Once a baseline length had been selected by one family, this option was not available to future enrollees. In turn, families who were enrolled later had fewer options to choose from and the final family was assigned a specific baseline length after other baseline lengths had been chosen. Caregivers completed daily baseline measures assessing parent-reported social anxiety symptoms and speaking behaviors, starting on the day of the pre-treatment session, and ending on the day of the first treatment session (i.e., Session 1).

#### 2.2.3. Treatment Phase

Following baseline data collection, all participants received 16 sessions of IBTSM, which were scheduled initially as weekly appointments in the community-based clinic. Caregivers continued to complete weekly questionnaires assessing their child’s social anxiety and speech across settings. Due to unexpected circumstances (e.g., illness, weather condition) treatment length ranged from 16 weeks to 22 weeks (M = 19 weeks), though one family (i.e., Child 3) completed all 16 sessions in 16 weeks. The condensed (i.e., 16-session) version of IBTSM maintained the same structure and key components of IBTSM in its original 20-session, 24-week format [10], only reducing the number of initial and intermediate exposure sessions (see Table 2). To facilitate appropriate in-session exposure practices, two sessions for Child 5 (Sessions 11 and 12) transitioned from the office to a naturalistic setting (e.g., at a local restaurant, at a playground).

#### 2.2.4. Project Personnel

Three doctoral-level students in school psychology completing advanced practicum experiences in the same community-based clinic served as treating clinicians. All had received training on common behavioral techniques typically used to treat children with SM from their direct clinical supervisor, who is a licensed psychologist with expertise in treating SM. An additional training session was held by the primary investigator/study coordinator to review IBTSM and its structure, to explain their roles and responsibilities with the project, and to model the observation procedures.

All study procedures and personnel were approved by the Biomedical Institutional Review Board at Michigan State University (Study 00000110, approved 10 April 2018).

### 2.3. Measures

#### 2.3.1. Treatment Adherence

Adherence checklists were developed for each session based on session descriptions/goals listed in the IBTSM manual [10]. Immediately following each session, study clinicians self-reported the extent to which they implemented each session component using a four-point scale, with options ranging from 0 to 3. Specific item-level responses included “No attempt was made”, “Attempted but not successful”, “Attempted and partially successful” and “Successful”. Percentages of adherence were derived from item-level responses after coding them into dichotomous No/Yes categories. A score of 0 or 1 (“no attempt” or “unsuccessful attempt”) was coded as “No” while a score of 2 or 3 (“partially successful attempt” or “successful attempt”) was coded as “Yes”. The number of components coded as “Yes” was divided by the total number of required components to yield an adherence percentage for that session.

#### 2.3.2. Baseline to End-of-Treatment Measures

Due to the behavioral conceptualization upon which IBTSM was developed, it is likely to be most effective for children with the “exclusively anxious” subtype of SM as described by Cohan and colleagues [17]. Participants needed to first meet criteria for the exclusively anxious SM clinical profile through confirmation of the absence of oppositional-anxious or anxious-language impaired subtypes. Currently, there are no validated approaches to subtype determination described in the SM literature. For the purposes of this study, subtype determination used multiple assessments of speaking behaviors, anxiety symptoms, aggressive behaviors, and history of speech delay.

#### 2.3.3. The Rule-Out Assessment

Using Cohan and colleagues’ [17] recommendation for refined methods to assess for SM subtypes, the Behavioral Concerns Inventory (BCI) and a comprehensive intake interview were used to rule out the presence of characteristics associated with other potential subtypes of SM (e.g., mildly oppositional/sensitive anxious SM, bilingual/communication-delayed anxious SM). The BCI is a 28-item checklist used to help caregivers indicate specific concerns they have about their child’s behavior. This list contains items describing various internalizing and externalizing behaviors. These included “Argues a lot”, “disobeys parents”, “Fights with other students”, and “Takes things that don’t belong to him/her”. If a caregiver reported two or more of these behaviors on the BCI, the child’s presenting symptoms would not be considered exclusively anxious SM. Additionally, a comprehensive clinical intake interview was administered during the pre-treatment intake session to gather information about the child’s developmental history, medical history, and current diagnoses to confirm the absence of a speech delay.

#### 2.3.4. The Social Anxiety Scale for Children—Revised (SASC-R)

Caregivers completed the Social Anxiety Scale for Children—Revised, Parent Form (SASC-R, Parent Version) [21] at pre-treatment and post-treatment. The SASC-R, Parent Version has no cut-off score to denote clinical significance, though the accompanying manual states that school-age boys receiving a score at above 50 and school-age girls with a score at or above 54 indicate high levels of social anxiety [21,22]. As a part of the clinical profile assessment, children eligible for treatment were required to demonstrate a total SASC-R score at or above a 50 (boys) or 54 (girls). The full caregiver form demonstrates adequate reliability in samples of children with SM (α = 0.87–0.91) [14].

#### 2.3.5. The Selective Mutism Questionnaire

Caregivers also completed the Selective Mutism Questionnaire (SMQ) [20] at baseline and again at the end of treatment to assess the frequency of their child’s speech across three contexts: Home, School, and Other/Public. Although there is no cut-off score for the SMQ indicating clinical severity, Bergman and colleagues [20] report a mean total score of 12.99 (SD = 7.23) for children with a primary diagnosis of SM. The full SMQ scale has adequate discriminant and convergent validity [20], and the most recent investigation of IBTSM revealed a Cronbach’s alpha of α = 0.78 [14].

#### 2.3.6. The Anxiety Disorders Interview Schedule (ADIS-P)

The Anxiety Disorders Interview Schedule for Children, Parent Version (ADIS-P) [23] was administered at baseline and at the end of treatment to assess diagnostic status for all five cases. The ADIS-P is a comprehensive structured interview schedule used to measure a child’s symptoms of anxiety and the extent to which these anxious symptoms interfere with their functioning. Caregiver responses yield a clinical severity rating (CSR) for each anxiety disorder. A CSR at or above four, on a 0–8 scale, indicates clinical significance [23]. The ADIS-P demonstrates good reliability (κ = 0.65–0.88; [24] and validity [25].

#### 2.3.7. The Screen for Child Anxiety Related Disorders (SCARED)

Caregivers also completed the Screen for Child Anxiety Related Disorders—Parent about Child Version (SCARED) [26] at baseline and at the final treatment session. The SCARED is a brief, broadband measure used to assess the frequency and severity of various symptoms in children including symptoms associated with social phobia/anxiety, school avoidance, panic disorder, generalized anxiety, and separation anxiety. Birmaher and colleagues [26] report high internal consistency for the 41-item measure, reporting an approximate reliability of α = 0.90.

### 2.4. Multiple-Baseline Measures

#### 2.4.1. Social Anxiety

Daily during the baseline phase and weekly during the treatment phase, caregivers were asked to complete the eight-item Fear of Negative Evaluation (FNE) subscale from the SASC-R to capture participants’ social anxiety symptoms related to perceptions of peer evaluation. As with the full scale, there are no clinical cut-offs for subscales on the SASC-R but Ginsburg et al. [27] report a mean of 23.30 (SD = 9.15) on the FNE subscale for socially anxious school-age children. The FNE subscale high reliability (α = 0.86) [21] and represented moderate severity (i.e., neither highest nor lowest mean scores) in a previous study of IBTSM for children with SM in a community-based setting, suggesting adequate sensitivity to potential treatment effects over time [15].

#### 2.4.2. Observed Speaking Behaviors

Two measures of observed speaking behaviors were used to track incremental changes across baseline and treatment phases for all five cases of children with exclusively anxious SM. First, caregivers completed the Brief Rating of Observed Speaking Behaviors (BROSB) every day during baseline and once per week during treatment to monitor incremental changes in speaking behaviors. The BROSB is a Direct Behavior Rating (DBR) [28] created specifically for the present study consisting of three items, derived from the three subscales of the SMQ [20]: home, school, and other social situations. Each item asks caregivers to rate the frequency and type of their child’s speaking behaviors for a given day/week using a seven-point Likert scale. Higher scores on the BROSB indicate increased frequency and complexity of speech. Additionally, analog behavioral observations (ABOs) were used to supplement caregiver ratings of speaking behaviors and to obtain quantitative data on speech frequency. As a part of each treatment session, the child, his or her caregiver, and the treating clinician engaged in a five-minute analog activity that encouraged speech using a pre-developed protocol. Each ABO took place before each of the 16 treatment sessions but the final ABO for session 16 took place during the final five minutes of the last treatment session. Clinicians tallied the number of words spoken during each ABO.

#### 2.4.3. Treatment Acceptability

The Treatment Evaluation Questionnaire (TEQ) [29] was used to gather input about caregivers’ satisfaction with IBTSM at the end of treatment. The TEQ contains 21 items across three subscales assessing Acceptability (11 items), Effectiveness (8 items), and Time Required (2 items). The TEQ was adapted into a questionnaire format from the Treatment Evaluation Inventory [30], which has high internal consistency α = 0.97. A summary of the assessment plan highlighting when each of the dependent measures were completed is presented in Table 3.

### 2.5. Data Analyses

#### 2.5.1. Treatment Adherence

Treatment adherence was analyzed by computing percentages of session components implemented by the study clinicians throughout IBTSM, per clinicians’ self-report. At the end of treatment overall, adherence percentages from one pre-treatment session and 16 active treatment sessions were summed and averaged. Individual adherence percentages were generated for each of the five cases, as well as an aggregated average percentage across all participants. Four in-clinic sessions for each participant were randomly selected for observation (live or via videotape) by the primary investigator to assess inter-observer agreement for overall adherence. Inter-observer agreement, calculated using the percentage of agreement between clinicians and the first author, was 94.2%.

#### 2.5.2. Treatment Effectiveness

Multiple-baseline measures. Visual analyses were used to determine the presence of noticeable changes in each individual child’s social anxiety levels and observed speaking behaviors over time, using guidelines for analyzing and reporting single-case intervention data [31]. The primary investigator and one project assistant used visual analysis guides to review three graphs for each child (i.e., SASC-FNE, BROSB, and ABO data) based on level, trend, variability, and immediacy of effect within a given case, as well as the consistency of patterns across all five cases. Agreement between visual analysis reviewers was adequate (κ = 0.60; 80% agreement). Disagreement only occurred when considering the immediacy of effect. Next, Tau-U effect size indices were used to assess the statistical significance of individual changes in outcome variables over time, and to assist in determining whether baseline trends were significant enough to require correction when examining these treatment effects. An online program was used to calculate Tau-U indices using both SASC-FNE and BROSB scores, for each individual child http://www.singlecaseresearch.org/calculators/tau-u (accessed on 5 April 2019) [32]. If a baseline-to-baseline contrast was considered statistically significant using a more liberal criterion for statistical significance (*p* < 0.15), as was recommended Bruni and colleagues [33], contrasts between baseline and treatment phase data controlled for baseline trend.

#### 2.5.3. Baseline to End-of-Treatment Measures

Reliable Change Index (RCI) [34] scores were used to assess clinical significance in observed changes on measures used at pre-treatment and end-of-treatment time points only (i.e., SASC-R, SMQ, and SCARED full scale). An RCI greater than 1.96 or less than −1.96 indicates a clinically significant change.

#### 2.5.4. Treatment Acceptability

Finally, treatment satisfaction was assessed through caregiver ratings on the TEQ-P for each child. An overall score of 110 or higher was used as an indicator of adequate treatment acceptability, while individual caregiver ratings of 55, 36, and 9 or higher were used to indicate adequate satisfaction across the Effectiveness, Acceptability, and Time Required subscales, respectively [35].

## 3. Results

### 3.1. Treatment Adherence

Clinicians’ self-report ratings of adherence to session components indicated high adherence when a condensed, 16-session version of IBTSM was implemented in a community-based clinical setting, as reflected in adherence ratings of 95% or higher across participants and an average adherence rating of 97% across all five children.

### 3.2. Treatment Effectiveness

#### 3.2.1. Multiple-Baseline Measures

**Figure 1:**
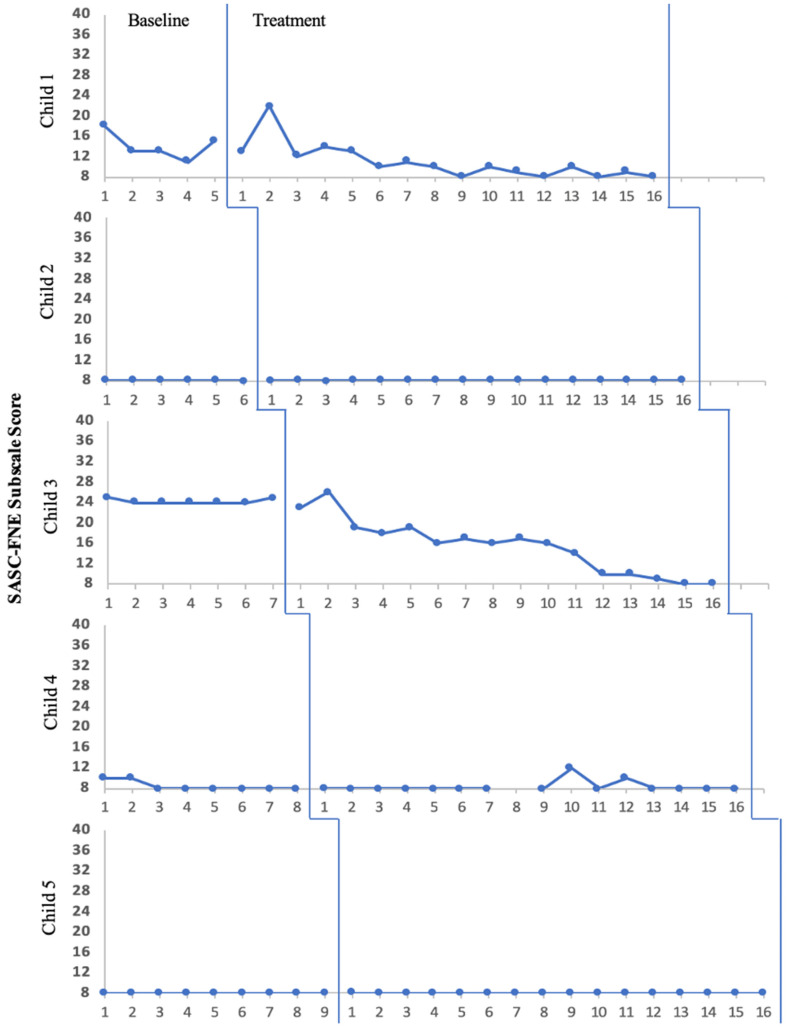
Caregiver-Rated Social Anxiety Levels Over Time.

**Figure 2:**
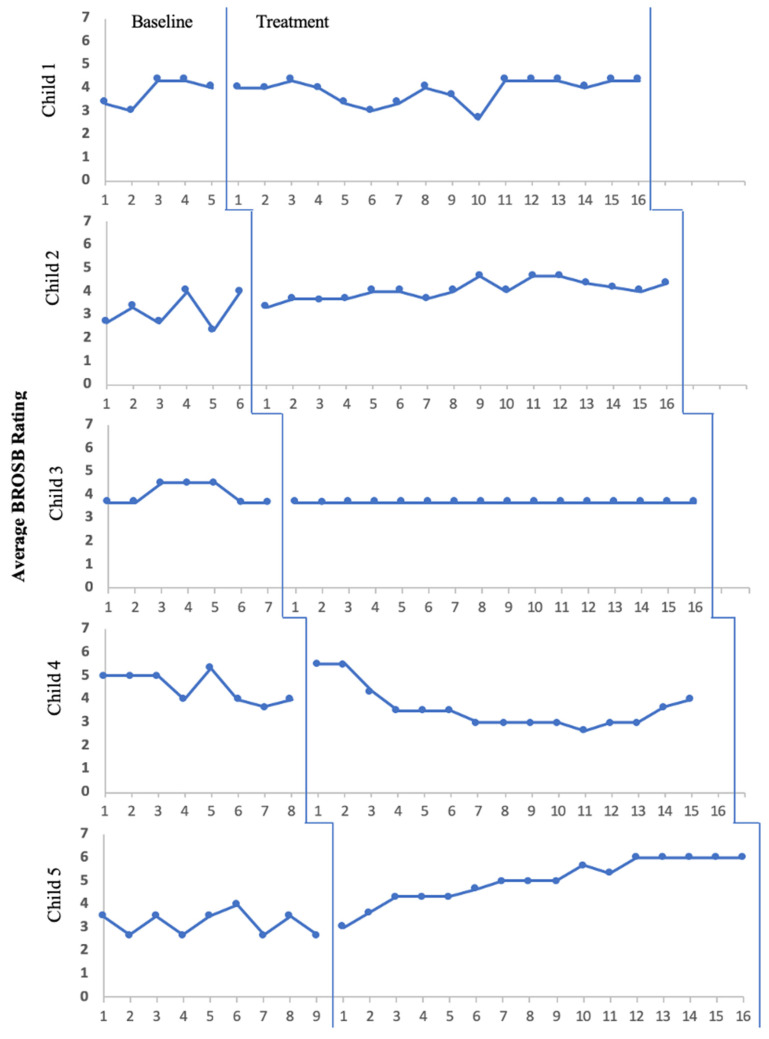
Average Scores on Brief Ratings of Observed Speaking Behaviors over Time.

Visual analyses for social anxiety levels (Figure 1) and speaking behaviors (Figure 2) did not provide evidence of a replicated treatment effect for reduced social anxiety symptoms or speaking behaviors across all five children, primarily due to a lack of change in level (i.e., mean) and trend (i.e., slope) in the anticipated direction following the introduction of the intervention. Of note, data presented in Figure 2 suggest that all five children demonstrated some level of comfort with speaking across settings (i.e., home, school, social/public) even prior to active treatment. Finally, simple visual analysis did not reflect noticeable, consistent improvement of words spoken during ABOs across the five participants.

Treatment effects were examined for individual participants using Tau-U effect size calculations. Using the cut-off of *p* < 0.15 as an indicator of statistical significance [33], two children experienced a significant reduction in parent-rated social anxiety symptoms over time (Child 1: Tau-U = −0.638, *p* = 0.035; Child 3: Tau-U = −0.875, *p* = 0.001). Additionally, only Child 2 and Child 5 experienced statistically significant increases in parent-rated speaking behaviors (Child 2: Tau-U = 0.677, *p* = 0.017; Child 5: Tau-U = 0.917, *p* < 0.001).

#### 3.2.2. Baseline to End-of-Treatment Measures

Based on changes in the SASC-R over time, four of five (80%) children saw a significant reduction in social anxiety symptoms (Child 1: RCI = −7.99; Child 3: RCI = −11.82; Child 4: RCI = −2.24; Child 5: RCI = −10.22). Supplemental assessments of child anxiety symptoms using the SCARED—Parent Form showed a similar pattern. Comparison of baseline targeting generalized anxiety or separation anxiety symptoms (Table 4). All five children saw an increase in SMQ scores from baseline to end of treatment, indicating an increase in caregiver-rated speaking behaviors over time. Three of five children (60%) were found to experience clinically meaningful increases in SMQ scores between baseline and end of treatment (Child 1: RCI = 5.42, Child 4: RCI = 4.34; Child 5: RCI = 6.23). By the end of treatment, three children (60%; Child 1, Child 4, Child 5) also saw a reduction in ADIS-P CSR scores for SM, reflecting a removal of diagnosis (Table 5).

### 3.3. Treatment Acceptability

A majority of caregivers (n = 4; 80%) perceived the condensed version of IBTSM as an acceptable treatment approach overall. All five (100%) caregivers endorsed adequate treatment quality, and all (100%) viewed the condensed version of IBTSM as acceptable regarding the time required. Caregivers of three participants (60%) perceived treatment to be adequately effective for their child.

## 4. Discussion

Treatment for SM typically utilizes evidence-based approaches developed for youth with social anxiety disorder [1]. To date, the most common and effective forms of treatment for children with SM based on meta-analytic techniques utilized with randomized controlled studies are behavioral approaches integrated with parent and teacher psychoeducation [9]. Some additional support exists for modular cognitive behavioral therapy involving psychoeducation, physiological training, cognitive training, behavioral training, parent training, and psychoeducational training for additional caregivers [7]. Although a number of studies have supported the effectiveness of various adaptations of behavioral or cognitive behavioral therapy for SM [12,36], no manualized treatment has garnered sufficient research support to be considered an EBT for SM [19].

The present study sought to contribute to this growing body of research on EBTs for children with SM, using a nonconcurrent multiple-baseline single-case design to examine the adherence, effectiveness, and acceptability of. This study builds upon previous investigations of the only manualized treatment for children with SM, Integrated Behavior Therapy for Selective Mutism (IBTSM) [10], which provide some preliminary evidence for its efficacy [14] and its potential effectiveness in reducing speech avoidance and social anxiety levels in children with SM in real-world contexts using varying treatment lengths [15,16]. Given limitations of previous studies (e.g., lack of sufficient baseline data, unclear assessment of intervention adherence, comorbid behavioral or speech/language deficits), a more rigorous single-case investigation was warranted in order more confidently draw conclusions about treatment effectiveness in specific populations of children with SM (i.e., children with an exclusively anxious subtype). Additionally, in an effort to better examine for whom IBTSM might be effective, this study investigated this manualized approach for children presenting with an exclusively anxious subtype of SM considering the possibility that there are distinct subtypes of SM, which may warrant different adaptations to behavioral therapy [13,17,36].

Five children between the ages of four and eight received a 16-session version of IBTSM across an average of 19 weeks (range = 16–22 weeks) in a community-based clinical setting. Novice clinicians implemented this condensed version of IBTSM with excellent adherence (i.e., average = 97%). This high treatment adherence seen across all five cases is consistent with previous investigations of IBTSM, which found similarly high ratings of treatment adherence when carried out in a research-based clinical setting (99.3%) [14] and a community-based clinical setting (96.7%) [15]. Additionally, caregivers uniformly perceived the condensed, 16-session version of IBTSM as acceptable. These results are in line with previous investigations of IBTSM when implemented in various settings and with varied treatment lengths [14,15].

Contrary to the hypothesized outcome, there was no evidence of a replicated treatment effect on social anxiety levels across all five of participants using visual analyses. However, four of five (80%) children saw clinically reliable reductions over time as indicated by RCI calculations, and two of the three children who met diagnostic criteria for social phobia at baseline saw a removal of diagnosis following 16 sessions of IBTSM over the course of 21 (Child 1) and 19 weeks (Child 4). Clinically meaningful reductions in social anxiety symptoms were also observed at the individual level in Siroky and colleagues’ [15] replicated AB single-case design and at the group level in the pilot RCT of IBTSM [14].

Visual analyses of caregiver ratings of speaking behaviors (i.e., BROSB) and words spoken during weekly analog behavioral observations did not support the hypothesis that all five children would experience a significant reduction in speech avoidance. When examining changes in SMQ total scores from baseline to end of treatment, however, it is clear all five children experienced an increase in speaking behaviors over time. Three children (60%) improved to the point of clinical significance, no longer meeting diagnostic criteria for SM by the end of treatment. This remission rate is comparable to the response Bergman and colleagues [14] found in the randomized-controlled pilot study of IBTSM where 67% of children in the treatment group saw a removal of diagnosis after 20 sessions of IBTSM.

An unexpected but critical finding in this study was the presence of relevant comorbid issues (e.g., other anxiety disorders, oppositional behaviors) and heterogeneity amongst the study sample, even after targeted efforts to identify and enroll children with a more homogenous clinical presentation of exclusively anxious SM. Within this sample of five children, three (60%) met criteria for one or more additional anxiety disorders beyond SM at baseline (Child 1: social phobia, GAD, separation anxiety; Child 2: social phobia; Child 4: social phobia, GAD). Although there is no research to date specifically examining how comorbid anxiety disorders affect treatment outcomes for children with SM receiving IBTSM, studies exploring predictors of treatment outcomes for children receiving CBT for anxiety suggest that child demographic variables at baseline (e.g., increased age, comorbid social anxiety, and greater symptom severity) may be associated with poorer treatment outcomes [37]. Contrary to those previous findings, however, two participants (i.e., Child 1 and 4) saw a removal of all baseline anxiety disorder diagnoses, including SM, by the end of treatment even though treatment did not specifically target symptoms of other anxiety disorders (e.g., separation anxiety, GAD).

Additional consideration should be given to the emergence of oppositional behaviors as seen within two participants (Child 2 and 3). One possible explanation for this change in clinical presentation, despite a demonstrated lack of oppositional behaviors in these two children at baseline, may reflect the changing patterns of parent responses to their child’s avoidance strategies when given the expectation to speak. The presence of oppositional behaviors in children with SM is not uncommon and believed to be associated with efforts to avoid or escape anxiety-provoking situations or obligations [13]. As children’s anxiety increases when faced with increased expectations for speaking, it is common for parents or caregivers to negatively reinforce avoidance through “rescuing” (i.e., speaking on behalf of the child) or removing the expectation to speak altogether [11]. In turn, parents may also develop a pattern of assisting their child in avoiding having to speak through reinforcement of oppositional behaviors. Over the course of IBTSM, these caregiver response patterns must be corrected by clinicians to reinforce the child’s speech, rather than speech avoidance.

## 5. Limitations

A critical consideration when reflecting on the lack of replicated treatment effects across all five cases is the selection of multiple-baseline measures to capture changes in dependent variables over time. Since caregivers were asked to provide ratings of social anxiety levels and speaking behaviors each week, there was a need to identify brief measures which caregivers could feasibly complete without compromising validity and reliability. Despite these efforts, the brief measures used in the present study may have been insufficient in adequately describing individual patterns of speaking behaviors and social anxiety symptoms for each child at baseline and, in turn, resulted in limited sensitivity to changes over the course of treatment.

The BROSB was a Direct Behavior Rating (DBR) developed specifically for this study, which was derived from the SMQ [20] as a brief way for caregivers to indicate the type of speech their child demonstrates at home, in school, and in other social situations. Although DBRs are supported as useful formative assessment tools sensitive to incremental change over time [38,39], there have been no previous studies utilizing DBRs for speaking behaviors in children with SM. Thus, it is possible that the BROSB as a tool was insufficient in capturing changes in individual’s speaking behaviors from week to week. For instance, during summer and winter breaks, caregivers were unable to rate their child’s speaking behaviors in the school domain, which resulted in decreased total scores on the BROSB. As a result, average item-level scores were used to better assess children’s speech across settings over the course of treatment. The use of means, rather than total scores, yielded a smaller range within which to improvements could be seen (i.e., 1–7 compared to 1–21).

Similarly, a proposed reason for a lack of change on the multiple-baseline measure of social anxiety (i.e., SASC-FNE subscale) is the unexpectedly low levels even at baseline, leaving minimal room for improvement over time. Using the screening procedures, all five children presented with clinically elevated social anxiety symptoms at baseline via SASC-R total scores. However, when caregivers provided daily ratings on the FNE subscale during the baseline phase, at least three children (Child 2, Child 4, Child 5) presented with minimal scores, leaving little room for improvement or observed change over the course of treatment. The FNE subscale was selected as the multiple-baseline assessment due to its high level of reliability (α = 0.86) [21], yet results from this investigation put into question the overall face validity of this subscale for use with children with SM.

Another potential limitation of the present study involves the procedure for identifying clinical subtypes of SM. The choice to screen for children with an exclusively anxious subtype of SM was based on Siroky and colleagues’ [15] findings, which suggested IBTSM may be specifically beneficial to children with that clinical presentation, rather than children with preexisting externalizing behaviors or speech/language concerns. Although preliminary research suggests the presence of at least three subtypes of SM [17], there are currently no empirically based assessments for identifying SM subtypes. In turn, conclusions about the effectiveness of IBTSM for the exclusively anxious subtype of children with SM are limited by unexpected changes in clinical presentations over the course of treatment in some, but not all, participants.

A final potential limitation of this study is the different intervals between data points for the baseline phase (one day), as compared to the treatment phase (one week). The baseline and treatment phases were designed in this way to reduce the amount of time children had to wait prior to the start of treatment, and to support the feasibility of data collection for participating caregivers. Although such a change in intervals between phases is not explicitly a concern identified by the single-case research design standards [31], this inconsistency may threaten the internal validity of the design and should be taken into consideration when interpreting results.

## 6. Implications for Research

Given a number of significant findings related to the adherence, effectiveness, and acceptability of a 16-session version of IBTSM when implemented in a community-based clinic, results from this study build upon previous investigations of IBTSM to better determine for whom and under which circumstances this treatment can be effective. Additional research exploring IBTSM’s effectiveness is needed to see if significant treatment effects can be replicated in new settings or with more diverse samples of children with SM.

First, an important feature of IBTSM is its emphasis on caregiver involvement throughout treatment, with increased responsibility as treatment progresses (i.e., “transfer of control”). It is unclear, however, the extent to which caregiver participation and perceptions contribute to IBTSM’s effectiveness. Even with this emphasis on caregiver skill training, the present study did not monitor the extent to which caregivers attempted and/or completed out-of-session exposure practices in other settings beyond the participating clinic. Future investigations of IBTSM should consider more closely examining the effect of caregiver involvement on treatment outcomes, including caregivers’ understanding of treatment processes/goals, engagement during treatment sessions, and adherence to out-of-session assignments.

Additionally, there is a clear need for the identification and clarification of possible subtypes of SM. Multiple efforts (e.g., developmental history checklist, diagnostic interview with parents) were made during the initial recruitment phase to identify children with an exclusively anxious subtype of SM, since it was hypothesized that other subtypes may show less improvement through the condensed version of IBTSM. However, based on clinician report and observation, at least two children were identified as having potential oppositional/defiant tendencies over the course of treatment. In turn, it is unclear which subtype would best capture the clinical presentation of these two children given the emergence of more externalizing behaviors over time. Efforts should be made through future research to develop a reliable and valid assessment for SM subtypes, with implications for treatment modifications to address distinct needs for those children with SM and their families.

Future studies would likely be strengthened by a more detailed examination of speaking behaviors in children with SM, including speech complexity, speech across settings, and possible safety behaviors specific to SM (e.g., speaking in a whisper or altered voice). It is well known that children with SM often experience more severe speech avoidance in the school setting as compared to other settings [2,3]. Previous investigations of IBTSM when implemented in a clinical research setting [14] and a community-based clinic setting [15] assessed treatment outcomes as rated by both parents and teachers. Unfortunately, it was beyond the scope of the present study to closely examine teacher-rated speaking behaviors.

Additionally, alternative measures may be helpful in assessing changes to specific patterns of speaking behaviors over the course of treatment. For instance, since the completion of the present study, Gensthaler and colleagues [40] developed a novel parent rating scale created specifically for children with SM, titled the Frankfurt Scale of Selective Mutism (FSSM). The FSSM includes two scales including a diagnostic scale, which is used to assess the presence of SM symptoms, as well as a severity scale to assess individual speaking behaviors, patterns, and the severity of speech avoidance. Xu and colleagues [41] also recently reviewed two pilot studies using passive audio vocal measurement (AVM) to provide more objective data regarding speaking behaviors over time. The use of AVM was found to be feasible and sensitive in capturing variation in vocalizations, which may help to provide greater detail about individual characteristics of each child’s pattern of communication.

## 7. Implications for Practice

The results of this study may also be used to inform clinical practice when treating children with SM. One finding from this investigation points to the potential feasibility of maintaining high treatment adherence while carrying out IBTSM in a community-based clinic setting. Three novice clinicians were able implement a condensed IBTSM (i.e., 16 sessions over an average of 19 weeks) with high levels of adherence to the session components as outlined in the manual. This adherence was also maintained across all five cases of children with SM, each presenting with varied goals related to speaking behaviors with peers, at school, or in other social situations in public. In the context of intervention research, such a finding is pertinent to more clearly examine the effectiveness of a specific treatment when carried out in novel settings. Further exploring IBTSM adherence, effectiveness, and acceptability by novel clinicians working under supervision of an expert supervisor is important not only within additional clinic-based settings, but also within school-based settings where children with SM often struggle.

Finally, results from this study provide some support for the presence of multiple subtypes of SM and the need for different treatment approaches to specific address clinical features of each subtype. All five participants presented with symptoms of the proposed exclusively anxious SM subtype at the baseline time point; however, two children displayed oppositional/defiant behaviors over the course of the active treatment phase. Although it may be challenging to predict which children will see an onset of oppositional behaviors once treatment begins, clinicians treating children with SM would do well to monitor early signs of defiance or oppositional behaviors (e.g., noncompliance with parental commands to engage in nonspeaking behaviors). This step may be critical in developing an effective and efficient treatment plan for individual children and to determine whether adaptations to IBTSM may be needed.

## 8. Conclusions

The present study sought to contribute to the growing body of research on EBTs for children with SM, using a nonconcurrent multiple-baseline single-case design to examine the adherence, effectiveness, and acceptability of the only manualized treatment for children with SM, Integrated Behavior Therapy for Selective Mutism [10]. This study builds upon previous investigations of IBTSM, which provide some preliminary evidence for its efficacy [14] and its potential effectiveness in reducing speech avoidance and social anxiety levels in children with SM in real-world contexts using varying treatment lengths [15,16].

Five children between the ages of four and eight with an exclusively anxious presentation of SM received a 16-session version of IBTSM in a community-based clinical setting. Novice clinicians implemented this condensed version of IBTSM with excellent adherence. Incremental assessment of social anxiety levels and speaking behaviors over the course of treatment indicated that each child experienced some individual improvement; however, visual analyses did not result in a replicated treatment effect across all five cases. Despite this lack of replicated effect using visual analyses, a majority of participants experienced statistically significant and clinically reliable reductions in SM symptoms, as demonstrated by Tau-U effect size calculations and RCI scores. More notably, three of five children no longer met diagnostic criteria for SM by the end of treatment and two children (40%) also saw a removal of comorbid anxiety disorder diagnosis by the same time point. Finally, caregivers rated this condensed version of IBTSM as acceptable overall. All five caregivers (100%) perceived IBTSM as acceptable with regard to treatment quality and time required. These findings suggest that IBTSM, when condensed into a 16-session version for use with novice clinicians in a community-based clinic, may be effective in significantly reducing primary symptoms of SM in children with the exclusively anxious subtype.

## Figures and Tables

**Table 1:** Baseline Demographics by Participant.

	Age	Sex	Race	ADIS-P, Clinical Severity Rating (CSR)				SMQ
				Selective Mutism	Social Phobia	GAD	Separation Anxiety	Total Score at Baseline
Child 1	8	M	Biracial	8	8	5	5	16
Child 2	7	M	Caucasian	8	5	n/a	n/a	13
Child 3	6	F	Caucasian	4	n/a	n/a	n/a	17
Child 4	6	M	Caucasian	6	6	7	n/a	16
Child 5	4	F	Caucasian	7	n/a	n/a	n/a	15

Note: ADIS-P = Anxiety Disorders Interview Schedule for Children, Parent Version. n/a = not applicable. On the ADIS-P, a CSR of 4 or higher indicates severity meeting clinical significance.

**Table 2:** Integrated Behavior Therapy for Selective Mutism Sessions: Comparison of Original and Condensed Versions.

IBTSM Sessions (24 Weeks, 20 Treatment Sessions Total)		Condensed IBTSM Sessions (M = 19 Weeks, 16 Treatment Sessions)	
Pre-treatment (Parent Only)	Assessment and Psychoeducation	Pre-treatment Intake Session	Assessment and Psychoeducation
Session 1	Introduction, Rapport Building	Session 1	Introduction, Rapport Building
Session 2	Rapport Building, Reward System, Feelings Chart	Session 2	Rapport Building, Reward System, Feelings Chart
Session 3	Classroom Chart, Fear Ladder, Exposure Practice	Session 3	Classroom Chart, Fear Ladder, Exposure Practice
Sessions 4–9	Initial Exposure Sessions	Sessions 4–7	Initial Exposure Sessions
Session 10	Midpoint Session	Session 8	Midpoint Session
Sessions 11–14	Intermediate Exposure Sessions	Sessions 9–10	Intermediate Exposure Sessions
Session 15	Exposure, Introduction to Transfer of Control	Session 11	Exposure, Introduction to Transfer of Control
Sessions 16–17	Exposure, Additional Transfer of Control	Sessions 12–13	Exposure, Additional Transfer of Control
Sessions 18–19	Exposure, Transfer of Control, Progress Review	Sessions 14–15	Exposure, Transfer of Control, Progress Review
Session 20	Relapse Prevention and Graduation	Session 16	Relapse Prevention and Graduation

**Table 3:** Overview of Assessment Plan by Phase.

Study Phase	Assessment Plan	Variable
Intake	Behavioral Concerns Inventory	Clinical profile of SM
	Clinical Intake Interview with Developmental and Medical History	
	Social Anxiety Scale for Children—Parent Version (Full scale)	Social anxiety severity
	Selective Mutism Questionnaire (Full scale)	SM symptom severity
	Anxiety Disorders Interview Schedule for Children—Parent Interview Screen for Child Anxiety Related Disorders—Parent Version	Clinical diagnoses
Baseline/ Treatment	Once weekly: Analog observations	Words spoken
	Once weekly: Session checklists	Treatment adherence
	Daily during baseline, Weekly during treatment: Social Anxiety Scale for Children—Parent Form (FNE subscale)	Caregiver-reported social anxiety levels
	Daily during baseline, Weekly during treatment: Brief Ratings of Observed Speaking Behaviors	Caregiver-reported speaking behaviors across contexts
	Four times per case: Direct observation of treating clinicians	Treatment adherence (inter-observer agreement)
End of treatment	Treatment Evaluation Questionnaire Social Anxiety Scale for Children—Parent Version (Full scale) Selective Mutism Questionnaire (Full scale) Anxiety Disorders Interview Schedule for Children—Parent Interview Screen for Child Anxiety Related Disorders—Parent Version	Treatment acceptability Social anxiety severity SM symptom severity Clinical diagnoses

**Table 4:** Changes in Anxiety Symptoms and Diagnoses from Baseline to End of Treatment.

Measure/Child		Baseline	End of Treatment	RCI
SASC-R Total Score				
Child 1		58	33	−7.99 *
Child 2		56	51	−1.60
Child 3		64	27	−11.82 *
Child 4		46	39	−2.24 *
Child 5		67	35	−10.22 *
**Average (SD)**		**58.20 (8.14)**	**37.00 (8.94)**	−6.71 *
SCARED Total Score				
Child 1		35	15	−4.29 *
Child 2		27	19	−1.72 *
Child 3		40	17	−4.93 *
Child 4		35	23	−2.57 *
Child 5		26	17	−2.57 *
**Average (SD)**		**32.60 (5.94)**	**17.60 (3.58)**	−3.22 *
ADIS-P—Social Phobia CSR				
Child 1		8	n/a	-
Child 2		5	6	-
Child 4		6	n/a	-
ADIS-P—GAD CSR				
Child 1		5	n/a	-
Child 4		7	n/a	-
ADIS-P—Separation Anxiety CSR				
Child 1		5	n/a	-
Child 4		n/a	4	-

Note. SASC-R = Social Anxiety Scale for Children, Revised; SCARED = Screen for Child Anxiety Related Disorders; ADIS-P = Anxiety Disorders Interview Schedule for Children, Parent Version; CSR = Clinical Severity Rating. n/a = not applicable * An RCI greater than 1.96 or less than −1.96 indicates a clinically significant change.

**Table 5:** Changes in Speaking Behaviors from Baseline to End of Treatment.

Measure/Child		Baseline	End of Treatment	RCI
SMQ Total Score				
Child 1		16	36	5.42 *
Child 2		13	18	1.36
Child 3		17	24	1.90
Child 4		16	32	4.34 *
Child 5		15	38	6.23 *
**Average (SD)**		**15.40 (1.52)**	**29.60 (8.41)**	3.85 *
ADIS-P—Selective Mutism CSR				
Child 1		8	n/a	-
Child 2		8	6	-
Child 3		4	4	-
Child 4		6	n/a	-
Child 5		7	n/a	-

Note. SMQ = Selective Mutism Questionnaire; ADIS-P = Anxiety Disorders Interview Schedule for Children, Parent Version; CSR = Clinical Severity Rating. n/a = not applicable * An RCI greater than 1.96 or less than −1.96 indicates a clinically significant change.

## Data Availability

Contact the first author for access to the data. The data are not publicly available due to ethical and legal limitations of confidentiality and privacy of protected health information.
